# Application of Cypermethrin-Coated ZnS and ZnO Nanoparticles against *Rhipicephalus* Ticks

**DOI:** 10.3390/pathogens12060807

**Published:** 2023-06-06

**Authors:** Tean Zaheer, Rao Zahid Abbas, Nighat Perveen, Olivier Andre Sparagano, Shanza Rauf Khan, Tauseef Ur Rehman, Ali Raza, Muhammad Kasib Khan, Muhammad Imran Arshad

**Affiliations:** 1 Department of Parasitology, University of Agriculture, Faisalabad 38000, Pakistan; 2 Department of Biology, College of Science, United Arab Emirates University, Al Ain P.O. Box 15551, United Arab Emirates; 3 Department of Infectious Diseases and Public Health, Jockey Club College of Veterinary Medicine and Life Sciences, City University of Hong Kong, Kowloon, Hong Kong SAR, China; 4 Department of Chemistry, University of Agriculture, Faisalabad 38000, Pakistan; 5 Department of Parasitology, Faculty of Veterinary and Animal Sciences, Islamia University Bahawalpur, Bahawalpur 63100, Pakistan; 6 Institute of Microbiology, University of Agriculture, Faisalabad 38000, Pakistan

**Keywords:** *Rhipicephalus*, control, cypermethrin-coated nanoparticles, zinc

## Abstract

*Rhipicephalus* ticks are described as important ticks impacting the costs of livestock rearing and by-products sale. The prevalence and response of ticks towards cypermethrin sprays indicate the need to implement the rational use of acaricides. In our previous studies, ZnO nanoparticles were shown to inhibit the major life-cycle stages of *Hyalomma* ticks, indicative of promising application of nanomaterials against the hard ticks. The current study was designed to probe into one of alternative options to curtail *Rhipicephalus* ticks by employing cypermethrin-coated nanoparticles of ZnO (C-ZnO NPs) and ZnS (C-ZnS NPs). The nanocomposites showed a roughly spherical type of morphology and various size dimensions upon characterization using SEM and EDX. Female ovipositioning was declined up to only 48% in ZnS and up to 32% in ZnO NPs even after 28 days in vitro. Similarly, the larval hatching was also impacted, leading to a hatching percentage of 21% and 15% by application of C-ZnS NPs and C-ZnO NPs, respectively. The LC_90_ in female adult groups were 3.94 mg/L and 4.27 mg/L for the C-ZnO NPs and C-ZnS NPs groups, respectively. Similarly, the larval groups had LC_90_ of 8.63 and 8.95 mg/L for the C-ZnO NPs and C-ZnS NPs groups. The study is a proof of the concept for incorporating effective and safe nanocomposites as acaricides. The studies on the efficacy and spectrum of non-target effects of nanomaterial-based acaricides can further refine the research on finding novel alternatives for tick control.

## 1. Introduction

*Rhipicephalus* ticks are economically important ticks of bovines, acting as vectors of protozoal (babesiosis and theileriosis), bacterial (anaplasmosis), viral (Thogoto), and rickettsial diseases [1]. Similarly, the diseases of ovine (Nairobi sheep disease, babesiosis, theileriosis, anaplasmosis, and rickettsiosis) present challenges for their effective prevention and control [2]. Approximately 80% of the cattle is at risk of contracting cattle ticks and the diseases that they vector, contributing to the overall tick- and tick-borne-disease-associated losses of up to USD 22–30 billion per annum [3]. Ticks are important vectors of animal diseases, influencing the production and performance parameters at the farm level. Owing to the inadequate application of acaricides, the ticks are showing resistance towards commonly used commercial preparations of acaricides. The scenario is anticipated to be worse in small herds in developing countries [4]. The aspect of drug residues within the environment, off-target organisms, and animal by-products is very crucial and often overlooked in the farmer communities that lack awareness.

*Rhipicephalus microplus* is the main cattle tick in developing countries [5]. The presence of resistant *Rhipicephalus* populations have been described worldwide and associated with significant livestock and public health problems [6]. In other developing countries including Brazil, India, Mexico, and Colombia, several studies have been undertaken, showing the resistance and/or vulnerability to organ-phosphorated and organ-chlorinated compounds, pyrethroids (deltamethrin, cypermethrin, flumethrin, alpha-cypermethrin, and lambda-cyhalothrin), methyl carbamate, and, recently, ivermectin, as well as others [7]. Keeping in view the importance of ticks and tick-borne diseases, several control strategies have been adopted for the control of ticks and associated diseases [8]. Recently, nanoparticles have gained currency, presenting a promising candidature for cattle tick control in the form of acaricides or tick repellents and vaccines [9,10]. Chemical control of ticks is the most widely used strategy worldwide. Owing to the non-judicious use of chemical acaricides (chemicals killing mites and ticks), the ticks of livestock have acquired different levels of tolerance and resistance [11]. The unguarded application of endectocides and some pesticides intended for tick control have further worsened the scenario. Cypermethrin and deltamethrin are among the widely recommended acaricides at the farm’s scale [12]. Ticks are developing resistance against these two chemicals at a steady pace. Therefore, it is high time that we devise some safe alternative yet efficient forms of these compounds to reduce the risks of developing resistance against current concentrations and forms of these acaricides. In this context, the current study was planned as a pioneer approach against *Rhipicephalus* ticks of large ruminants, featuring the application of nanomaterials of a metallic nature and combinations with cypermethrin. In addition to this, the cytotoxicity of cypermethrin-coupled ZnO nanoparticles and cypermethrin-coupled ZnS nanoparticles were also estimated.

## 2. Materials and Methods

### 2.1. Collection and Identification of Ticks

We have chosen livestock-rich areas of Punjab province, which are also diverse ecological zones. The convenient sampling method was applied to collect ticks from livestock animals including cattle and buffalo. A total of 410 ticks were collected and 100 adult ticks were subjected to bioassays. The subsequent egg masses laid were further utilized for egg immersion tests. Ticks from the ears, lips, eyelids, and tails of animals were collected in vials with no added chemicals for nanoparticle assays. The bottles had small holes (inflicted with common pins) in the lids of the collection bottles. These perforations allowed aeration to keep the ticks active until the incubation/bioassays. The stereomicroscope-assisted taxonomy of ticks was performed following the guidelines of Walker and co-workers [13]. The tick anatomical features of the mouth parts, basis capitulum, coxa, scutum, and other features were assessed for identification at the genus level. The taxonomic identification was performed to only include the ticks of *Rhipicephalus* ticks (regardless of their sex) in the current study.

### 2.2. Synthesis of Zinc Sulfide Nanoparticles

Zinc acetate dihydrate (Zn(CH_3_COO)_2_ × 2H_2_O), thiourea (N_2_H_4_CS), polyethylene glycol (C_16_H_34_O_9_), urea (CO(NH_2_)_2_), and ammonia (14 *v*/*v*% NH_3_) were purchased from Sigma Aldrich (St. Louis, MO, USA). All the chemicals were used as received. First, 35 mL of 0.04 M zinc acetate dihydrate and 0.04 M thiourea were magnetically stirred for 1 h and shifted into a Teflon chamber kept in stainless-steel-lined autoclave. The reaction was carried at 170 °C for 1 h. After 1 h, the autoclave was cooled to room temperature and the product was collected by centrifugation at 3000 rpm. Product was washed with water three times to obtain neutral pH and dried at 80 °C for 4 h.

### 2.3. Synthesis of Zinc Oxide Nanoparticles

First, 1.5 g zinc acetate dehydrate, 1.5 mL polyethylene glycol, 1 g urea, and 6.5 mL ammonia were dissolved in 65 mL distilled water, and pH is maintained at 10. The reaction mixture was heated at 110 °C for 5 h in Teflon chamber sealed in stainless-steel-lined autoclave. The product was collected by centrifugation and washed three times with water. The product was dried at 80 °C for 4 h and ground to fine powder.

### 2.4. Coating of ZnS and ZnO Nanoparticles with Cypermethrin

The same procedure was used for coating of insecticide cypermethrin on both ZnO and ZnS nanoparticles. First, 1 g nanoparticles and 10 mL water were magnetically stirred at room temperature. Polyvinyl pyrollidene was added pinch by pinch until a colloid was formed. On the other side, 32.5 mg cypermethrin was dissolved in 10 mL of water. Both the solutions were mixed and stirred for 24 h at room temperature. The obtained product was centrifuged at 3000 rpm for 30 min to separate nanoparticles coated with cypermethrin.

### 2.5. Tick Bioassays

The efficacy of zinc oxide nanoparticles (ZnO NPs), zinc sulphide nanoparticles (ZnS NPs), cypermethrin-coated ZnO NPs (ZnO-Cyp NPs), cypermethrin-coated ZnS NPs (ZnS-Cyp NPs), and cypermethrin was evaluated in *Rhipicephalus* ticks. Various concentrations of nanoparticles (3 mg/L, 6 mg/L, 9 mg/L, and 12 mg/L) were applied on each of life stage of tick, i.e., egg, larval, and adult stages. The following tests were included in this study:

EIT: The Egg Immersion Test;

LIT: Larval Immersion Test;

AIT: Adult Immersion Test.

The bioassays were performed in an aerobic incubator with 80 to 90% relative humidity and temperature range of 28 to 30 °C. The duration of post-treatment observations consisted of 24 to 48 h for adult males and larvae. On the other hand, owing to the days taken for ovipositioning stage of *Rhipicephalus* ticks, the observation time was kept up to 15 days [14,15,16]. Cypermethrin was tested at 0.001, 0.01, 0.1, and 1%. Different dilutions were made from the stock solutions which contained 100× of the dilution proposed by the manufacturer of commercially available cypermethrin for use in veterinary field. The brief details about the trial are as follows.

#### 2.5.1. Egg Immersion Test

The tests were executed on eggs obtained from ovipositioning females after 3–5 days of egg laying. About 200 eggs (approximately weighing 0.01 g) were placed in 500 µL of test acaricide for 5 min. The eggs were treated for each concentration and at least three replicates of each test concentration (3 mg/L, 6 mg/L, 9 mg/L, and 12 mg/L) were used. The test solutions were decanted off and tubes containing treated eggs were allowed to dry [17]. The test tubes were closed with cotton plugs and incubated at 27–28 °C at 70–80% relative humidity. Egg laying and hatching into larvae (hatching%) were the criteria for estimation of nanoparticle efficacy within observation time of 15 days and after 15 days:

(1) $$Hatching\;\%=\frac{Number\;of\;hatched\;larvae}{Total\;number\;of\;eggs\;incubated}$$

where this ratio indicates mortality of eggs as well as inability of eggs to hatch into larval stages.

#### 2.5.2. Larval Immersion Test

The larval ticks (at least 7 days old) were put to larval immersion test as per protocol proposed by the FAO [14]. The ticks were immersed in 10 mL of various concentrations of acaricides and NPs. Further procedures were same as mentioned above to apply different concentrations of nanoparticles on ticks and to find mortality. The ticks were randomly selected and treated for each concentration and at least three replicates were used.

#### 2.5.3. Adult Immersion Test (AIT)

Adult ticks were processed for Adult Immersion Test using protocols proposed by FAO (2004) [14] with slight modifications. The ticks were kept immersed in 10 mL of acaricides and NPs at various concentrations. Further protocols were same as described above to find percentage mortality of ticks’ post 24 h incubation. The sexed ticks were randomly selected and treated for each concentration and at least three replicates were used. Abbot’s formula was followed for mortality calculation:

(2) $$Corrected\;Mortality=\frac{\%\;treated\;mortality-\%\;control\;mortality}{100-\%\;control\;mortality}\times 100$$

### 2.6. Changes in Tick Morphology

Tick morphology including size, margins, and hardness of their scutum were checked through a stereomicroscope. The treated groups were compared with the control group for morphological appearances. The darkening of cuticle layers of ticks indicated death of the ticks. However, engorged ticks found without egg mass around them also indicated death of the ticks. Further declaration of death of the ticks was performed by finding no response to the blow/prick.

### 2.7. Toxicity Evaluation—Plant Model: Allium cepa Ana-Telophase Test

The idea of choosing any study model for nanotoxicity revolves around the potential of that model organism to represent the non-target parts of eco-system. For this study, the onion model was utilized for estimation of ecotoxic and genotoxic effects of nanomaterials proposed in this study. Similar models based on animals or cell lines could also be explored to estimate potential genotoxicity. *Allium cepa* test was performed for various concentrations of nanoparticles and acaricides using guidelines of Liman et al., 2022 [18]. Briefly, small onions were submerged in distilled water, negative control, and different preparations (1.25 mg/mL, 2.5 mg/mL, and 5 mg/mL) of treatments for 48 h. Slides of onion roots were evaluated under microscope and photographed while 500 to 550 cells were evaluated for mitotic index using following formula:

(3) $$MI=\frac{Number\;of\;cells\;in\;division}{Number\;of\;total\;cells}\times 100$$

(4) $$Phase\;index=\frac{Particular\;phase}{Number\;of\;cells\;in\;division}\times 100$$

### 2.8. Comet Assay on A. cepa Root Tips

DNA damage was determined by protocols defined by Liman [18,19], with some modifications made for comet assay. Various solutions were made, and, adopting above referenced protocols, DNA damage ranging 0–4 was classified based on integrity of head and length of tail. Using arbitrary units, the following formula was used to assess DNA damage:

(5) $$Arbitrary\;Unit=\sum_{i=0}^{4}Ni\times i$$

*Ni* = number of cells;

*I* = degree of damage (0–4).

### 2.9. Data Analysis

The LC_50_, LC_90_, and associated confidence intervals were estimated using the mortality data by applying Probit analysis by SPSS version 22 of statistical program. ANOVA was applied to determine the statistical differences among treatment groups. Parametric and non-parametric tests were applied at 5% probability.

## 3. Results

The nanoparticle yield by the hydrothermal method used in this study was around 90% for all NP groups. The size of ZnS–cypermethrin-coated nanoparticles lies around 1–2 µm. These spherical particles were assembled from smaller nanoparticles whose size lies around 20–100 nm. The size of cypermethrin-coated ZnO nanotubes lies around 0.8 to 6.0 µm. These nanotubes were formed by the spiral assembly of small nanoparticles ranging 20–100 nm in size.

### 3.1. SEM Image Analysis of Nanoparticles

**Figure 1:**
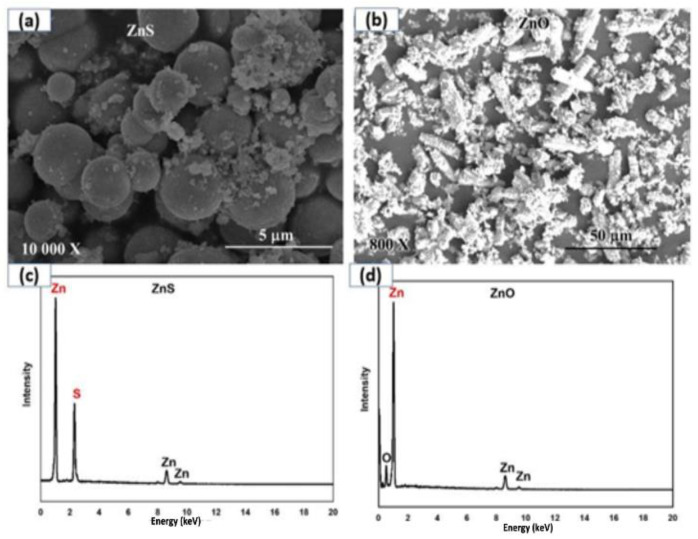
SEM analysis (**a**,**b**) and EDX characterization (**c**,**d**) of ZnO and ZnS nanoparticles.

The SEM images in Figure 1a exhibit ZnS NPs, which were synthesized by using the hydrothermal method, at different magnifications. The results were recorded across a range of magnifications from lower to higher. The external morphology of the product was analyzed by using SEM, and the images are presented in Figure 1. Figure 1a illustrates that the particles are agglomerated and lack sharp edges. In contrast, Figure 1b, which is a magnified version of (a), reveals the presence of a few needle-like particles. The figure reveals that the product is composed of tiny, tube-shaped, and flower-like particles. The tubes are well-separated and randomly oriented, with no aggregation in the product. At certain points, the nanotubes are attached side by side, forming layers of multichannel tubes.

### 3.2. EDX Patterns of Nanomaterials

The EDX pattern of the synthesized ZnS nanoparticles is shown in Figure 1c. The intense peaks appearing at 1.0 keV and 8.6 keV are characteristic of the Lα transition occurring from the M shell to the L shell (*n* = 3 → 2) of Zn. The peaks at 2.3 keV and 1.8 keV show that the electron transition occurs from the L shell to the K shell, which is characteristic of the Kα transition of the electron jumping from (*n* = 2 → 1) of Zn. Figure 1d shows the EDX pattern of synthesized ZnO nanoparticles. The intense peaks at 1.0 keV and 8.6 keV represent the Lα transition from the M shell to the L shell (*n* = 3→2) of Zn. The peaks at 0.8 KeV and 1.8 KeV indicate the electron transition from the K shell to the L shell, which is the characteristic of the Kα transition of the electron jumping from (*n* = 1 → 2) of Zn. The peak at 7.8 KeV is characteristic of the Kβ transition, indicating that the intensity of the Kα transition is greater than that of Kβ, and there is a higher probability of Kβ transition where the electron jumps from *n* = 3 of the “s” to the “p”.

### 3.3. Effect of Cypermethrin, ZnO, ZnS NPs, and Their Coated Nanoparticles on Ticks

**Figure 2:**
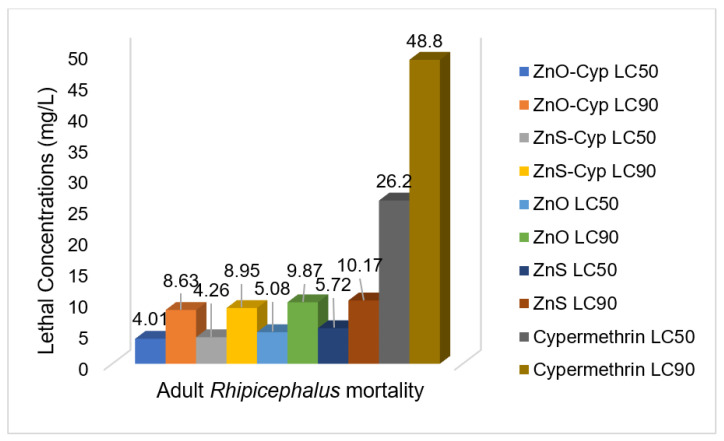
Lethal concentrations calculated by Probit analysis from adult tick bioassays.

**Figure 3:**
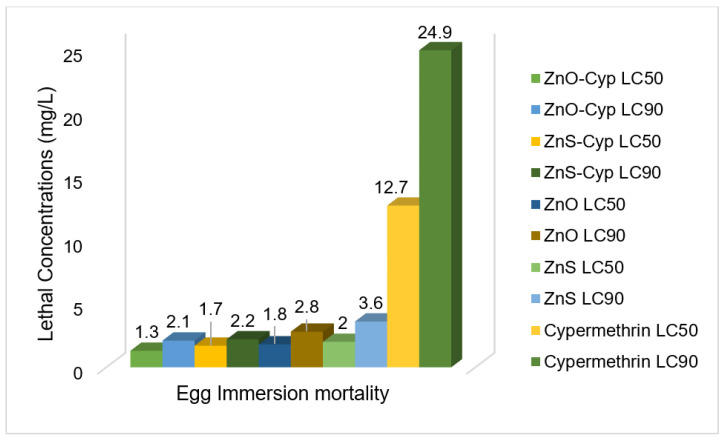
Lethal concentrations for egg stage of *Rhipicephalus* ticks calculated by Probit.

**Figure 4:**
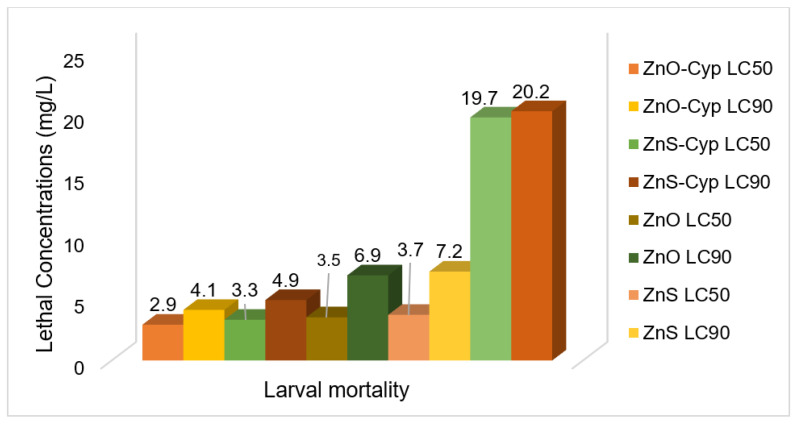
Lethal concentrations for larval stage of *Rhipicephalus* ticks calculated by Probit.

**Figure 5:**
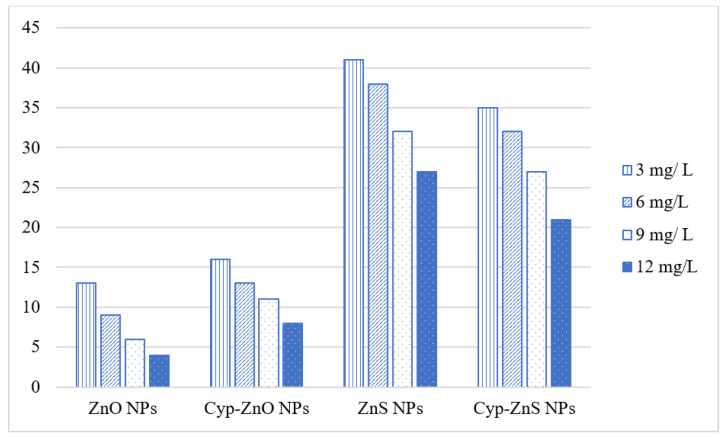
Ovipositioning owing to ZnO and ZnS nanoparticles alone and in combination with cypermethrin within 20 days of adult assay.

**Figure 6:**
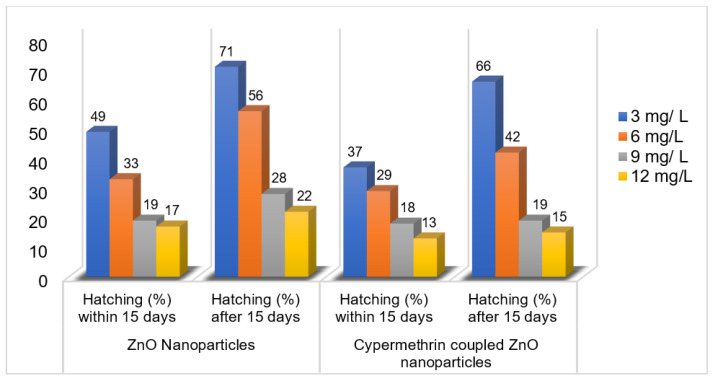
Percentage hatching of tick larvae against ZnO nanoparticles alone and in combination with cypermethrin within 15 days and after 15 days post-treatment.

**Figure 7:**
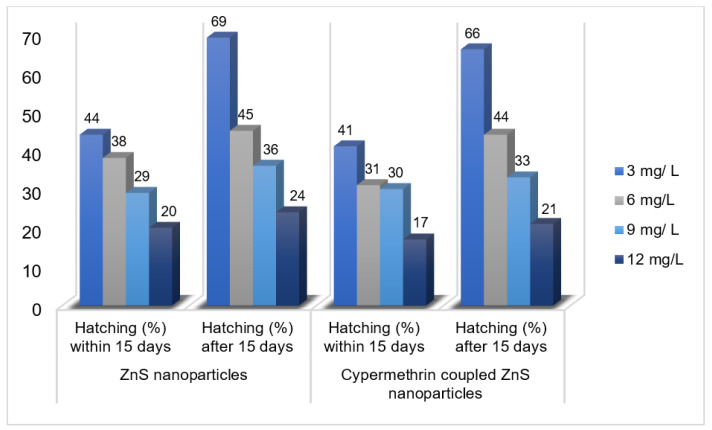
Percentage hatching of tick larvae against ZnS nanoparticles alone and in combination with cypermethrin within 15 days and after 15 days post-treatment.

Based on the convenient sampling, the species of *Rhipicephalus* (*R.)* collected from bovines were: *R. microplus*, *R. decoloratus,* and *R. evertsii*. The tick bioassays performed revealed lethal concentrations in adult, larval, and egg assays without standard deviations, respectively (Figure 2, Figure 3 and Figure 4). The ANOVA statistics revealed significant differences among different life-cycle stages of *Rhipicephalus* used in this study (Table 1). The ZnO nanoparticles coated with cypermethrin (LC_90_ = 8.63 mg/L) have shown better results among others where the ZnS coated with cypermethrin (LC_90_ = 8.95 mg/L) had lethal concentrations lower than the corresponding ZnS-alone (LC_90_ = 10.17 mg/L) treatment group. However, the cypermethrin treatment group showed higher lethal concentrations (LC_90_ = 48.8 mg/L) among all the treatment groups. The effect of uncoated and coated nanomaterials on ovipositioning in female ticks have been shown in Figure 5. Female ovipositioning was halted to 48% in ZnS-treated and up to 32% in ZnO-NPs-treated female ticks even after 28 days post-treatment. Similarly, the larval hatching was also impacted, leading to a lower hatching percentage of 21% and 15% compared to 92% of the control group (untreated group) by application of C-ZnS NPs and C-ZnO NPs, respectively (Figure 6 and Figure 7).

Taken together, the promise of Cyp-ZnO NPs against *Rhipicephalus* ticks in vitro was higher compared to Cyp-ZnS NPs. Similarly, the uncoated nanomaterials of ZnO showed lower lethal concentrations compared to the ZnS-NPs-treated group and the highest lethal concentrations were observed in the cypermethrin-treated group.

### 3.4. Changes in Tick Morphology

The ticks that died within 24 h of nanoparticle exposure appeared to be shriveled. Moreover, the ticks became reluctant to crawl/walk even when allowed to move in the Petri plates. This was confirmed by the 72 h post-exposure mortality data where the tibia of the ticks became brittle and fell off from the body. These changes indicated acute stress on the ticks owing to the nanoparticle exposure. The egg masses laid by nanoparticle-treated females became desiccated within.

### 3.5. Cytotoxicity and Genotoxicity of Different Preparations

The effect of different preparations (cypermethrin-coated ZnO and ZnS nanoparticles) on the mitotic and phase index in *A. cepa* roots and their effect on DNA damage in *A. cepa* root tips at different concentrations were observed. The composites showed responses that are non-significant with that of negative control, indicating these to be safe to use.

### 3.6. Effect of Cypermethrin

**Figure 8:**
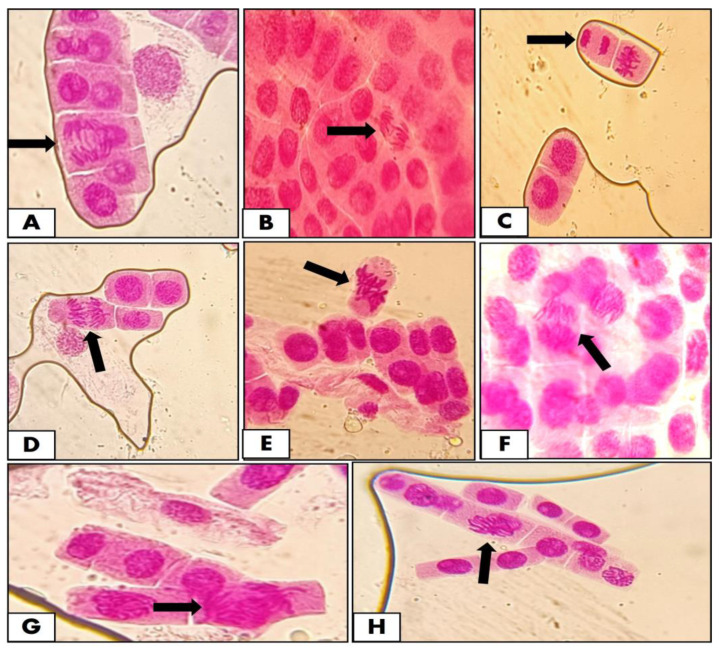
Chromosomal aberrations induced by various concentrations of Cyp, ZnS-coated Cyp, and ZnS-coated Cyp: (**A**) chromosomal bridge, (**B**) anaphase bridge, (**C**) stickiness, (**D**) polyploidy, (**E**) vagrant/chromosomal break, (**F**) mitosis, (**G**) laggard formation, and (**H**) interphase.

Cytotoxic and genotoxic effects were observed as a measure of the decrease in the mitotic index and mitotic phases after the application of cypermethrin (Figure 8). It was observed that DNA damage was increased with an increase in concentration of cypermethrin and its contact time with onion cells. The stickiness of chromosomes and metaphase abnormalities were observed due to the genotoxic effects of cypermethrin. Similarly, levels of DNA damage were also observed due to cypermethrin treatment as shown (Table 2, Table 3, Table 4 and Table 5).

ZnO-coated cypermethrin (Cyp) concentrations also showed low genotoxic and cytotoxic effects. The highest value of phase index was observed for 1 mg/mL ZnO-coated Cyp (163 ± 1.26, 72 h). In the case of DNA damage, concentration-dependent DNA damage was observed; the highest level of DNA damage was observed in 1 mg/mL ZnO-coated Cyp (166 ± 1.19, 72 h).

### 3.7. Effect of Cypermethrin-Coated ZnS Nanoparticles

No significant cytotoxic and genotoxic effects of the cypermethrin-coated ZnS nanoparticles were observed compared to the negative control. A time- and concentration-dependent increase in the mitotic index and mitotic phases were observed compared to the control. Similarly, a time- and concentration-dependent decrease in DNA damage was observed by the cypermethrin-coated ZnS nanoparticles on onion root tips compared to the control (Table 6 and Table 7).

The ZnS-coated cypermethrin and ZnS-coated cypermethrin showed slightly genotoxic and cytotoxic effects. In the case of the ZnS-coated cypermethrin, a moderate level of DNA damage was observed. The highest level of DNA damage was induced in the concentration (1 mg/mL; 162.4 ± 1.26) for 72 h.

Since Cyp-coated ZnS had mild genotoxic effects, chromosomal bridges, breaks, and c-mitosis were the chromosomal aberrations observed. From a toxicity analysis, it can be presumed that the nanoparticles of ZnO and ZnS were found to be having minimum to mild toxicity effects as observed by chromosomal aberrations and DNA damage in the onion model. Similarly, the cypermethrin-coated nanomaterials of ZnO and ZnS showed minimum to mild toxicity effects compared to the mild–moderate toxicity effects exhibited by cypermethrin alone.

## 4. Discussion

The *Rhipicephalus* ticks hold economic importance, having a broad coverage of hosts including mammals, reptiles, and avian species [20]. The parasite adaptation in *Rhipicephalus* ticks has made them successful parasites of public health importance [1]. *Rhipicephalus* ticks are known for high genetic diversity, enabling them to thrive in different geographical regions of the world [21]. Moreover, a study in Pakistan reported the presence of pathogens in *Rhipicephalus* ticks, having the potential of causing livestock and human diseases [22]. The control of ticks from the context of a developing nation becomes more challenging owing to the limited updates on epidemiology, farmer/herd owner awareness, and eagerness to adapt adequate tick control strategies [23]. Routinely, the commercially available acaricides are used to get rid of ticks whose inadequate dosage and regimes have led to the development of acaricide resistance in ticks [24]. Recently, a study from Pakistan has reported rapidly emerging resistance in ticks against routinely used acaricide formulations [25]. The GluCl receptor, which prevents drug binding to its target site, has been associated with ivermectin resistance in acari (spider mites) [26]. This scenario could signpost toward the rise of resistance, and, thus, the urgency of seeking alternatives or reproposing the existing drugs in promising forms.

Among alternatives to chemical acaricides, plant-based products and nanomaterials have gained research focus in the past few decades. The nanoparticles could be a promising tool against ticks and tick-borne pathogens owing to the fine particle size causing the oxidative stress and cellular injury in non-mammalian cells only [27]. Compared to widely used chemical acaricides, the process of nano-based acaricides may be more challenging [28]. This is partially due to the: (i) acceptability of conventional farmers in adopting a modern acaricide; (ii) costs incurred to rationalize the dose and routes of administration; (iii) labour and expertise intensiveness of nanomaterial synthesis; and (iv) study of nano-acaricides from a wider lens, contextualizing the ‘one-health approach’. However, these factors may not restrict the application of nanomaterials arising from pre-existing chemical acaricides. The world has recently witnessed their rapid commercialization, promising immunity, and people’s eagerness to adopt nano-vaccines against the COVID-19 pandemic [29].

The size of nanoparticles in the present study ranged from 80 to 200 nm, approximately. The SEM images further indicate that the as-synthesized ZnS nanoparticles exhibit a spherical morphology, are densely packed, and are polydispersed. However, Figure 1b highlights that the ZnS nanospheres formed are not transparent and possess a rough surface. Additionally, the particles are non-uniform in size [30]. The magnified view of the nanoflower demonstrates that the assembly of multichannel tubes, layer by layer, creates the nanoflower morphology, as in the figure. The different nanotubes stack together to form multichannel tubes, which then arrange themselves to form a nanoflower. Various ZnO nanomaterial morphologies have been reported, such as nanoflakes, nanorods, nanospheres, nanoneedles, nanowires, nanopowder, nanofilms, nanobelts, nanodiscs, nanoflowers, and nanotubes, among others. However, tubes and flowers have not been reported in any preparation technique so far. EDX patterns of nanomaterials revealed the electron transitions characteristic of Zn [31]. The peak at 7.8 keV is characteristic of the Kβ transition, which indicates that the intensity of the Kα transition is greater than that of Kβ. Therefore, there is a higher probability of the Kβ transition, and the electron jumps from *n* = 3 of the “p” to the “s” [32].

The nanoparticles were well-dispersed, round-to-cubical in shape with corners curved and smooth boundaries. The ZnO nanoparticles were practically spherical in form with smooth surfaces, according to previous research [33]. Some of the particles were found to be well-scattered, whereas the majority were clumped together, in coherence with other similar-natured nanomaterials [34]. Rapid reduction, assembly, and room-temperature sintering of spherical nanoparticles were used to create the observed nanostructures. Studies have reported the promising results of titanium dioxide nanoparticles (having LC_50_ = 5.43 mg/L), *Eucalyptus-globulus*-loaded nano-emulsions (controlling female reproduction up to 97.8%), and copper nanoparticles (having LC_50_ = 14.14 mg/L) against the adult stages of *R. microplus,* respectively [35,36,37]. Similarly, nickel nanoparticles (having LC_50_ = 10.17 mg/L), plant-based silver nanoparticles showing LC_50_ = 7.61 mg/L, and nanoparticles synthesized from cinnamon oil showing a maximum inhibition at 5% have been reported against the larval stages of *Rhipicephalus* ticks [38,39,40]. Owing to the fine particle size capable of both the up- and down-regulation of oxidative and cell injury pathways in ticks [27], the nanomaterials have shown enhanced acaricidal potential when compared to the herbal/chemical constituents alone.

The scenario of deltamethrin and ZnO NPs also revitalized the idea of repurposing already existing acaricides in more effective forms. The results revealed 100% and 70% mortality in deltamethrin and ZnO nanocomposite. Deltamethrin–silver nanoparticle groups were treated at 2 mL/L of the test concentration [28]. The same study reported the lack of efficacy of imidacloprid nanocomposites against *Rhipicephalus* ticks. The application of silica nanoparticles loaded with Spinosad led to an enhancement in skin adhesion and the subsequent efficacy of nano-bio-acaricide against cattle ticks [41]. The loading of silica nanoparticles and Spinosad brought a rough-surfaced material that itself encouraged the action of the target acaricide, in accordance with our results. The nanomaterials developed by a biological route had shown relatively lower LC_50_ compared with nanomaterials synthesized following chemical routes. This is evident from the study by Abdel-Ghany and co-workers, who reported an LC_50_ of 11.6 mg/mL in the egg immersion test compared with the lower (1.8) seen in our study [42]. In contrast to that, in our previous study, the LC_50_ attributed to the green-mediated ZnO nanoparticles of neem and lemon grass were lower for adults (4.76 and 4.92 mg/L, respectively) compared with LC_50_ of 5.03 mg/L in adults used for current study [43]. These differences in observations could be attributed to the repletion status of the ticks used for bioassays; moreover, the differences in days post-ovipositioning may also influence the results of EIT for the evaluation of nanotoxicity. An assay featuring the use of the same metal nanoparticle synthesized by both the chemical and biological route, having the same inclusion criteria for ticks/eggs, could be designed to compare the promise of both routes in tick control.

The results of DNA damage in *A. cepa* root tips treated with cypermethrin-exposed root tips showed significantly higher DNA damage than the negative control group. DNA damage and chromosomal aberrations were higher in the cypermethrin-alone group followed by the cypermethrin-coated ZnO and ZnS groups. According to Amaç and Liman, 2021; Liman et al., 2021, no statistically significant difference was observed between the positive control group and the 100 µg/L clopyralid (except at 24 h) concentrations [18,44]. The gradual increase in the CAs reveals the genotoxic effects of cypermethrin. According to Liman et al., (2021) [18] there was a concentration-dependent decrease in MI (r = −0.99) at all concentrations of WO_3_NPs compared to that of the negative control group. The negative control showed the highest MI value (74.67 ± 0.78), while the least (24.64 ± 0.72) was observed by the highest concentration of WO3NPs. The reduction in MI was even lower after the 12.5 mg/L compared to that of the positive control group. A dose-dependent increase in the mitotic phases was shown after the exposure of WO_3_NPs by all concentrations compared to the negative control group, except for the prophase.

## 5. Conclusions

There is an urgent need to address the emerging resistance in ticks against commonly used commercial acaricide preparations. This study provides a proof of concept to repurposing already existent drugs in a modified, yet safer manner. The toxicity analysis has shown mild effects owing to the use in the onion-toxicity model for nanoparticles. The study found that the ZnO NPs coating with cypermethrin led to better tick toxicity relative to the ZnS NPs coating with cypermethrin at the laboratory scale. Moreover, there is statistically significant difference among the NPs treatments given at the egg, larval, and adult stage of the ticks. Furthermore, it is required to probe into the application of these novel nanomaterials at the field scale. The accurate doses and the routes of nano-pesticides need a research focus to catalyze the process of their economical production and commercialization as seen in other nano-based commercial products round the globe.

## Figures and Tables

**Table 1:** LC_50_ and LC_90_ of different preparations against adult, egg, and larval stages of ticks.

Treatment Group	Adults	Eggs	Larvae
Lethal Concentration: LC_50_			
ZnO-Cyp	4.01 ± 0.11 ^a^	1.33 ± 0.51 ^a^	2.93 ± 0.25 ^a^
ZnS-Cyp	4.26 ± 0.09 ^a^	1.7 ± 0.2 ^a^	3.3 ± 30.5 ^a^
ZnO	5.08 ± 0.11 ^b^	1.8 ± 0.26 ^a^	3.5 ± 0.4 ^a^
ZnS	5.72 ± 0.16 ^b^	2.06 ± 0.21 ^a^	3.7 ± 0.26 ^a^
Cypermethrin	26.23 ± 0.57 ^c^	12.70 ± 0.26 ^b^	19.7 ± 30.32 ^b^
Lethal Concentration: LC_90_			
ZnO-Cyp	8.63 ± 0.11 ^a^	2.12 ± 0.33 ^a^	4.1 ± 0.1 ^a^
ZnS-Cyp	8.95 ± 0.06 ^ab^	2.19 ± 0.27 ^a^	4.93 ± 0.25 ^b^
ZnO	9.87 ± 0.21 ^bc^	2.8 ± 0.36 ^ab^	6.9 ± 0.26 ^c^
ZnS	10.17 ± 0.12 ^c^	3.6 ± 0.26 ^b^	7.2 ± 0.3 ^c^
Cypermethrin	48.8 ± 0.72 ^d^	24.9 ± 0.3 ^c^	20.23 ± 0.42 ^d^

Different superscripts within column indicate significant difference (*p <* 0.05).

**Table 2:** Effect of cypermethrin (Cyp) on phase index and DNA damage in *A. cepa* roots at different concentrations.

		Phase Index (%) ± SD				DNA Damage Mean ± SD
Concentration (mg/mL)	CCN	Prophase	Metaphase	Anaphase	Telophase	
**24 h**						
Control	500	103 ± 3.17 ^c^	5 ± 0.31 ^e^	7 ± 0.55 ^f^	8 ± 0.57 ^d^	48 ± 0.99 ^f^
Positive control	500	214 ± 3.14 ^d^	10 ± 0.91 ^d^	12 ± 0.975 ^e^	10 ± 0.91 ^cd^	145 ± 0.79 ^e^
0.0001 mg/mL Cyp	500	276 ± 3.21 ^a^	13 ± 1.01 ^c^	17 ± 1.356 ^cd^	13 ± 1.01 ^bc^	158 ± 0.81 ^d^
0.001 mg/mL Cyp	500	235 ± 3.13 ^b^	16 ± 1.36 ^b^	19 ± 1.49 ^bc^	15 ± 1.257 ^ab^	175 ± 0.19 ^c^
0.01 mg/mL Cyp	500	237 ± 3.22 ^b^	14 ± 1.20 ^bc^	21 ± 1.55 ^ab^	14 ± 1.20 ^ab^	181 ± 2.01 ^b^
0.1 mg/mL Cyp	500	235 ± 3.19 ^b^	19 ± 1.49 ^a^	15 ± 1.257 ^de^	17 ± 1.356 ^a^	186 ± 2.07 ^a^
1 mg/mL Cyp	500	268 ± 3.27 ^a^	9 ± 0.8 ^d^	23 ± 1.570 ^a^	16 ± 1.36 ^ab^	190 ± 2.11 ^a^
**48 h**						
0.0001 mg/mL Cyp	500	221 ± 3.11 ^c^	17 ± 1.36 ^bc^	11 ± 0.95 ^b^	9 ± 0.8 ^c^	196 ± 2.10 ^e^
0.001 mg/mL Cyp	500	234 ± 3.17 ^b^	15 ± 1.257 ^c^	12 ± 0.975 ^ab^	16 ± 1.36 ^a^	203 ± 2.13 ^d^
0.01 mg/mL Cyp	500	255 ± 3.09 ^a^	11 ± 0.95 ^d^	14 ± 1.20 ^a^	15 ± 1.257 ^a^	207 ± 2.17 ^c^
0.1 mg/mL Cyp	500	263 ± 3.257 ^a^	23 ± 1.570 ^a^	11 ± 0.95 ^b^	11 ± 0.95 ^bc^	214 ± 2.22 ^b^
1 mg/mL Cyp	500	213 ± 2.99 ^c^	19 ± 1.49 ^b^	13 ± 1.01 ^ab^	14 ± 1.20 ^ab^	219 ± 2.18 ^a^
**72 h**						
0.0001 mg/mL Cyp	500	230 ± 3.01 ^c^	24 ± 1.5 ^a^	19 ± 1.49 ^b^	17 ± 1.356 ^c^	225 ± 2.13 ^e^
0.001 mg/mL Cyp	500	232 ± 2.989 ^c^	13 ± 1.13 ^cd^	17 ± 1.36 ^bc^	21 ± 1.55 ^b^	231 ± 2.1 ^d^
0.01 mg/mL Cyp	500	242 ± 2.89 ^b^	17 ± 1.36 ^b^	25 ± 1.62 ^a^	27 ± 1.70 ^a^	238 ± 2.16 ^c^
0.1 mg/mL Cyp	500	257 ± 3.0 ^a^	11 ± 0.95 ^d^	14 ± 1.20 ^c^	14 ± 1.20 ^c^	244 ± 2.21 ^b^
1 mg/mL Cyp	500	253 ± 3.02 ^a^	15 ± 1.257 ^bc^	16 ± 1.36 ^bc^	16 ± 1.36 ^c^	251 ± 2.20 ^a^

Different superscripts within column indicate significant difference (*p* < 0.05). The analysis is performed separately for each of 24 h, 48 h, and 72 h.

**Table 3:** Effect of cypermethrin (Cyp) on chromosomal aberrations (CAs) in *A. cepa* roots at different concentrations.

CAs	CCN	0.0001 mg/mL Cyp	0.001 mg/mL Cyp	0.01 mg/mL Cyp	0.1 mg/mL Cyp	1 mg/mL Cyp
**24 h**						
Anaphase bridge	500	7 ± 0.55 ^c^	5 ± 0.31 ^d^	8 ± 0.57 ^bc^	9 ± 0.8 ^b^	11 ± 0.95 ^a^
Chromosomal laggard	500	5 ± 0.31 ^a^	2 ± 0.05 ^c^	6 ± 0.38 ^b^	3 ± 0.1 ^c^	9 ± 0.8 ^a^
Polyploidy	500	6 ± 0.38 ^a^	3 ± 0.02 ^d^	5 ± 0.31 ^b^	6 ± 0.38 ^a^	3 ± 0.8 ^c^
Stickiness	500	11 ± 0.95 ^bc^	9 ± 0.8 ^c^	13 ± 1.01 ^b^	16 ± 1.36 ^a^	12 ± 0.975 ^b^
C-mitosis	500	14 ± 1.20 ^c^	11 ± 0.95 ^c^	18 ± 1.45 ^b^	21 ± 1.55 ^ab^	23 ± 1.6 ^a^
Interphase	500	113 ± 2.01 ^a^	170 ± 1.9 ^a^	139 ± 1.91 ^b^	120 ± 1.89 ^c^	109 ± 2.5 ^d^
Chromosomal Bridge	500	11 ± 0.95 ^b^	8 ± 0.57 ^c^	9 ± 0.8 ^bc^	10 ± 0.91 ^bc^	14 ± 1.20 ^a^
Chromosomal break	500	14 ± 1.20 ^a^	7 ± 0.55 ^b^	16 ± 1.36 ^c^	9 ± 0.8 ^b^	3 ± 0.1 ^c^
**48 h**						
Anaphase bridge	500	5 ± 0.31 ^b^	2 ± 0.05 ^c^	6 ± 0.38 ^b^	3 ± 0.1 ^c^	9 ± 0.8 ^a^
Chromosomal laggard	500	9 ± 0.8 ^b^	11 ± 0.95 ^b^	14 ± 1.20 ^a^	11 ± 0.95 ^b^	9 ± 0.8 ^b^
Vagrant	500	-	-	-	-	5 ± 0.31
Polyploidy	500	8 ± 0.57 ^ab^	7 ± 0.55 ^bc^	9 ± 0.8 ^a^	9 ± 0.8 ^a^	6 ± 0.43 ^c^
Stickiness	500	11 ± 0.95 ^a^	17 ^b^	13 ± 1.122 ^cd^	24 ± 1.5 ^a^	15 ± 1.257 ^bc^
C-Mitosis	500	9 ± 0.8 ^a^	9 ± 0.8 ^a^	7 ± 0.55 ^a^	7 ± 0.55 ^a^	13 ± 1.13 ^a^
Interphase	500	168 ± 2.07 ^a^	144 ± 2.35 ^c^	124 ± 2.7 ^d^	97 ± 1.81 ^e^	161 ± 2.01 ^b^
Chromosomal Bridge	500	16 ± 1.36 ^a^	13 ± 1.12 ^abc^	12 ± 0.975 ^bc^	15 ± 1.257 ^ab^	11 ± 0.95 ^a^
Chromosomal break	500	13 ± 1.12 ^bc^	16 ± 1.36 ^ab^	17 ± 1.36 ^a^	18 ± 1.45 ^a^	12 ± 0.975 ^c^
**72 h**						
Anaphase bridge	500	7 ± 0.55 ^c^	4 ± 0.16 ^d^	9 ± 0.8 ^bc^	12 ± 0.975 ^a^	8 ± 0.57 ^bc^
Chromosomal laggard	500	11 ± 0.95 ^a^	3 ± 0.1 ^c^	2 ± 0.05 ^cd^	5 ± 0.31 ^b^	1 ± 0.01 ^d^
Polyploidy	500	5 ± 0.31 ^b^	11 ± 0.95 ^a^	3 ± 0.1 ^a^	12 ± 0.975 ^a^	5 ± 0.31 ^b^
Stickiness	500	13 ± 1.13 ^c^	16 ± 1.36 ^bc^	21 ± 1.33 ^a^	19 ± 1.49 ^ab^	18 ^ab^
C-mitosis	500	14 ± 1.20 ^bc^	19 ± 1.49 ^a^	13 ± 1.13 ^c^	2 ± 0.05 ^d^	17 ± 1.36 ^ab^
Interphase	500	139 ± 2.70 ^bc^	147 ± 2.57 ^a^	127 ± 2.7 ^d^	144 ± 2.66 ^ab^	133 ± 2.3 ^cd^
Chromosomal Bridge	500	12 ± 0.975 ^a^	7 ± 0.55 ^b^	6 ± 0.38 ^d^	9 ± 0.8 ^bc^	11 ± 0.95 ^ab^
Chromosomal break	500	9 ± 0.8 ^ab^	10 ± 0.91 ^a^	8 ± 0.57 ^bc^	1 ± 0.04 ^d^	7 ± 0.55 ^c^

Different superscripts within row indicate significant difference (*p* < 0.05). The analysis is performed separately for each of 24 h, 48 h, and 72 h.

**Table 4:** Effect of ZnO-coated cypermethrin on phase index and DNA damage in *A. cepa* roots at different concentrations.

CAs	CCN	0.0001 mg/mL ZnO-Coated Cyp	0.001 mg/mL ZnO-Coated Cyp	0.01 mg/mL ZnO-Coated Cyp	0.1 mg/mL ZnO-Coated Cyp	1 mg/mL ZnO-Coated Cyp
**24 h**						
Anaphase bridge	500	3 ± 0.02 ^e^	3.3 ± 0.02 ^d^	4 ± 0.03 ^c^	7 ± 0.18 ^a^	5 ± 0.06 ^b^
C-Mitosis	500	5 ± 0.06 ^a^	7 ± 0.18 ^d^	9 ± 0.185 ^c^	11 ± 0.20 ^a^	10 ± 0.19 ^b^
Chromosomal break	500	4.1 ± 0.03 ^d^	5 ± 0.06 ^c^	8 ± 0.16 ^b^	9 ± 0.185 ^a^	4 ± 0.03 ^d^
Chromosomal Bridge	500	4.5 ± 0.04 ^a^	4.9 ± 0.05 ^c^	5 ± 0.06 ^c^	8 ± 0.16 ^a^	7 ± 0.18 ^b^
**48 h**						
Chromosomal laggard	500	5 ± 0.06 ^e^	8 ± 0.16 ^d^	10 ± 0.19 ^c^	11 ± 0.20 ^b^	12 ± 0.22 ^a^
C-Mitosis	500	8 ± 0.16 ^e^	9 ± 0.185 ^d^	10.25 ± 0.191 ^c^	12 ± 0.22 ^b^	13 ± 0.231 ^a^
Anaphase bridge	500	10 ± 0.19 ^e^	12 ± 0.22 ^d^	13 ± 0.231 ^c^	14 ± 0.25 ^b^	15.3 ± 0.28 ^a^
Chromosomal Bridge	500	13 ± 0.231 ^d^	14 ± 0.25 ^c^	15 ± 0.27 ^b^	16.07 ± 0.3 ^a^	16.17 ± 0.31 ^a^
**72 h**						
Anaphase bridge	500	8 ± 0.02 ^b^	4 ± 0.03 ^d^	7 ± 0.18 ^c^	8 ± 0.16 ^b^	10 ± 0.19 ^a^
Chromosomal laggard	500	11 ± 0.20 ^e^	13 ± 0.231 ^d^	14 ± 0.25 ^c^	15 ± 0.27 ^b^	17 ± 0.35 ^a^
C-Mitosis	500	12 ± 0.22 ^c^	11 ± 0.20 ^d^	13 ± 0.231 ^b^	12 ± 0.22 ^c^	15 ± 0.27 ^a^
Chromosomal break	500	13 ± 0.231 ^d^	15 ± 0.27 ^c^	15.5 ± 0.29 ^c^	17 ± 0.35 ^b^	18 ± 0.4 ^a^
Chromosomal Bridge	500	15 ± 0.27 ^d^	18 ± 0.4 ^b^	19 ± 0.44 ^a^	13 ± 0.231 ^e^	17 ± 0.35 ^c^

Different superscripts within row indicate significant difference (*p* < 0.05). The analysis is performed separately for each of 24 h, 48 h, and 72 h.

**Table 5:** Effect of ZnO-coated cypermethrin (Cyp) on CAsin *A. cepa* roots at different concentrations.

		Phase Index (%) ± SD				DNA Damage Mean ± SD
Concentration (mg/mL)	CCN	Prophase	Metaphase	Anaphase	Telophase	
**24 h**						
Control	500	102 ± 1.17 ^f^	4 ± 0.12 ^e^	5 ± 0.4 ^b^	4 ± 0.11 ^d^	48 ± 0.69 ^c^
Positive control	500	114 ± 1.11 ^e^	5 ± 0.15 ^d^	5.2 ± 0.02 ^b^	4.23 ± 0.03 ^d^	146 ± 0.74 ^b^
0.0001 mg/mL ZnO-coated Cyp	500	118 ± 1.18 ^d^	5.6 ± 0.16 ^cd^	5.9 ± 0.03 ^a^	4.83 ± 0.07 ^c^	147 ± 0.79 ^b^
0.001 mg/mL ZnO-coated Cyp	500	121 ± 1.19 ^d^	6 ± 0.2 ^bc^	5.61 ± 0.13 ^ab^	4.95 ± 0.13 ^bc^	148 ± 0.21 ^b^
0.01 mg/mL ZnO-coated Cyp	500	127 ± 1.23 ^c^	6.6 ± 0.25 ^ab^	6.1 ± 0.19 ^b^	5.14 ± 0.151 ^bc^	152 ± 0.97 ^a^
0.1 mg/mL ZnO-coated Cyp	500	131 ± 1.19 ^b^	6.9 ± 0.29 ^a^	6.7 ± 0.21 ^b^	5.27 ± 0.16 ^b^	153 ± 1.03 ^a^
1 mg/mL ZnO-coated Cyp	500	138 ± 1.21 ^a^	7 ± 0.3 ^a^	7.2 ± 0.22 ^a^	6.3 ± 0.20 ^a^	153.1 ± 1.03 ^a^
**48 h**						
0.0001 mg/mL ZnO-coated Cyp	500	141 ± 1.1 ^d^	7.9 ± 0.36 ^a^	8 ± 0.24 ^c^	7 ± 0.3 ^c^	155 ± 1.01 ^c^
0.001 mg/mL ZnO-coated Cyp	500	143 ± 1.05 ^cd^	8 ± 0.4 ^a^	9 ± 0.25 ^c^	9.4 ± 0.26 ^b^	156 ± 1.09 ^bc^
0.01 mg/mL ZnO-coated Cyp	500	145 ± 1.01 ^bc^	8.22 ± 0.42 ^a^	9.3 ± 0.252 ^b^	9.71 ± 0.27 ^ab^	158 ± 1.12 ^abc^
0.1 mg/mL ZnO-coated Cyp	500	147 ± 1.13 ^b^	8.51 ± 0.50 ^a^	10 ± 0.26 ^a^	10 ± 0.31 ^ab^	159 ± 1.21 ^ab^
1 mg/mL ZnO-coated Cyp	500	150.6 ± 1.18 ^a^	9 ± 0.44 ^a^	9.8 ± 0.22 ^ab^	10.3 ± 0.33 ^a^	160 ± 1.15 ^a^
**72 h**						
0.0001 mg/mL ZnO-coated Cyp	500	153 ± 1.22 + c	10.6 ± 0.5 ^b^	10.4 ± 0.24 ^d^	10.25 ± 0.36 ^c^	162 ± 1.09 ^b^
0.001 mg/mL ZnO-coated Cyp	500	155 ± 1.18 ^c^	11 ± 0.54 ^b^	11 ± 0.26 ^cd^	11.4 ± 0.4 ^bc^	164 ± 0.96 ^ab^
0.01 mg/mL ZnO-coated Cyp	500	158 ± 1.08 ^b^	12 ± 0.62 ^ab^	11.5 ± 0.217 ^bc^	11.65 ± 0.6 ^bc^	164.3 ± 1.07 ^ab^
0.1 mg/mL ZnO-coated Cyp	500	161 ± 1.25 ^ab^	13 ± 0.63 ^a^	12 ± 0.19 ^ab^	11.8 ± 0.62 ^b^	165 ± 1.14 ^a^
1 mg/mL ZnO-coated Cyp	500	163 ± 1.26 ^a^	13.41 ± 0.69 ^a^	12.2 ± 0.30 ^a^	14 ± 0.7 ^a^	166 ± 1.19 ^a^

Different superscripts within column indicate significant difference (*p* < 0.05). The analysis is performed separately for each of 24 h, 48 h, and 72 h.

**Table 6:** Effect of ZnS-coated cypermethrin on phase index and DNA damage in *A. cepa* roots at different concentrations.

		Phase Index (%) ± SD				DNA Damage Mean ± SD
Concentration (mg/mL)	CCN	Prophase	Metaphase	Anaphase	Telophase	
**24 h**						
Control	500	103 ± 1.17	4.53 ± 0.21	5 ± 0.55	4 ± 0.47	48 ± 0.69
Positive control	500	114 ± 1.14	6 ± 0.31	5.75 ± 0.02	4.23 ± 0.01	145 ± 0.79
0.0001 mg/mL ZnS-coated Cyp	500	121 ± 1.22	6.2 ± 0.1	5.9 ± 0.03	4.83 ± 0.07	148.5 ± 0.81
0.001 mg/mL ZnS-coated Cyp	500	129 ± 1.14	6.9 ± 0.3	6.13 ± 0.49	4.95 ± 0.23	149.3 ± 0.19
0.01 mg/mL ZnS-coated Cyp	500	133 ± 1.20	7.1 ± 0.20	6.78 ± 0.53	5.14 ± 0.21	151 ± 1.01
0.1 mg/mL ZnS-coated Cyp	500	137 ± 1.18	7.4 ± 0.43	8 ± 0.51	5.27 ± 0.16	153 ± 1.05
1 mg/mL ZnS-coated Cyp	500	140.3 ± 1.25	7.98 ± 0.18	8.04 ± 0.57	6.3 ± 0.26	153.6 ± 1.052
**48 h**						
0.0001 mg/mL ZnS-coated Cyp	500	142 ± 1.01	8.13 ± 0.36	8.7 ± 0.59	9.1 ± 0.28	154.1 ± 1.06
0.001 mg/mL ZnS-coated Cyp	500	145 ± 1.07	9 ± 0.24	10 ± 0.67	9.4 ± 0.26	157 ± 1.13
0.01 mg/mL ZnS-coated Cyp	500	148.5 ± 1.1	10.5 ± 0.5	9.3 ± 0.62	9.71 ± 0.27	158.2 ± 1.14
0.1 mg/mL ZnS-coated Cyp	500	151 ± 1.15	11 ± 0.70	10.4 ± 0.65	10 ± 0.30	159 ± 1.21
1 mg/mL ZnS-coated Cyp	500	155 ± 1.39	11.6 ± 0.42	10.95 ± 0.61	10.3 ± 0.33	159.4 ± 1.17
**72 h**						
0.0001 mg/mL ZnS-coated Cyp	500	158 ± 1.21	11.66 ± 0.67	11 ± 0.49	10.25 ± 0.35	160 ± 1.13
0.001 mg/mL ZnS-coated Cyp	500	161 ± 1.18	13 ± 0.74	12 ± 0.69	11.4 ± 0.55	161 ± 1.1
0.01 mg/mL ZnS-coated Cyp	500	165 ± 1.8	14 ± 0.66	12.3 ± 0.7	11.65 ± 0.60	161.3 ± 1.15
0.1 mg/mL ZnS-coated Cyp	500	167 ± 1.40	12.8 ± 0.59	12.9 ± 0.80	11.8 ± 0.61	161.73 ± 1.20
1 mg/mL ZnS-coated Cyp	500	169 ± 1.6	14.4 ± 0.29	9 ± 0.64	14 ± 0.66	162.4 ± 1.26

**Table 7:** Effect of ZnS-coated cypermethrin (Cyp) on *A. cepa* roots at different concentrations.

CAs	CCN	0.0001 mg/mL ZnO-Coated Cyp	0.001 mg/mL ZnO-Coated Cyp	0.01 mg/mL ZnO-Coated Cyp	0.1 mg/mL ZnO-Coated Cyp	1 mg/mL ZnO-Coated Cyp
**24 h**						
Anaphase bridge	500	3 ± 0.02	4 ± 0.03	5 ± 0.06	5.4 ± 0.063	4 ± 0.03
C-Mitosis	500	6 ± 0.07	8 ± 0.14	7 ± 0.10	11 ± 0.81	9 ± 0.15
Chromosomal break	500	8 ± 0.14	7 ± 0.10	11 ± 0.81	6 ± 0.07	5 ± 0.06
**48 h**						
Chromosomal laggard	500	7 ± 0.10	9 ± 0.15	12 ± 0.975	11 ± 0.81	10 ± 0.17
C-Mitosis	500	8 ± 0.14	6 ± 0.07	9 ± 0.15	9 ± 0.15	7 ± 0.10
Anaphase bridge	500	11 ± 0.81	12 ± 0.975	14 ± 1.07	16 ± 1.09	15 ± 1.08
Chromosomal Bridge	500	14 ± 1.07	13 ± 1.0	12 ± 0.975	17 ± 1.091	16 ± 1.09
**72 h**						
Anaphase bridge	500	7 ± 0.10	5 ± 0.06	8 ± 0.14	12 ± 0.975	9 ± 0.15
Chromosomal laggard	500	10 ± 0.17	5 ± 0.06	3 ± 0.02	5 ± 0.06	6 ± 0.07
C-Mitosis	500	6 ± 0.07	10 ± 0.17	2 ± 0.028	12 ± 0.975	4 ± 0.03
Chromosomal break	500	9 ± 0.15	12 ± 0.975	11 ± 0.81	9 ± 0.15	13 ± 1.0

## Data Availability

There are no linked data for this article.
